# Epidemiological analysis of wild mushroom poisoning in Zhejiang province, China, 2016–2018

**DOI:** 10.1002/fsn3.2646

**Published:** 2021-10-29

**Authors:** Lili Chen, Liang Sun, Ronghua Zhang, Ningbo Liao, Xiaojuan Qi, Jiang Chen, Ting Liu

**Affiliations:** ^1^ Department of Nutrition and Food Safety Zhejiang Provincial Center for Disease Control and Prevention Hangzhou China; ^2^ College of Food Science and Engineering Jiangxi Agricultural University Nanchang China; ^3^ Institute of Remote Sensing and Earth Sciences Hangzhou Normal University Hangzhou China

**Keywords:** mushroom, poisoning, spatial analysis

## Abstract

Wild mushroom poisoning has been recognized as a global problem threatening human health. In this study, we aimed to explore characteristics of wild mushroom poisoning in Zhejiang province, China. From 2016 to 2018, 429 cases of wild mushroom poisoning were reported, and among them, there were 2 deaths and 84 hospitalizations, with the incidence of 0.2526 per 100,000 and the case fatality rate of 0.47%. Digestive symptoms were found in all cases. Systemic symptoms and signs, neurological symptoms, and urinary symptoms were found in 28.90% (124/429), 11.66% (50/429), and 4.90% (21/429) of the cases, respectively. The proportion of cases with incubation period <6 h was 85.78%, and those with ≥6 h accounted for 14.22%. The peak period of poisoning occurred from June to October annually. Quzhou (Moran’s *I* = 1.242, *p* < .05) and Lishui (Moran’s *I* = 0.759, *p* < .05) with mild climate, more mountains, and abundant rainfall were “hot spots” for the incidence of wild mushroom poisoning, showing a state of high‐incidence aggregation. Epidemiological analysis showed that there were seasonal, high‐incidence areas and high‐risk groups in wild mushroom poisoning. The government should give early warning to high‐incidence areas and strengthen publicity to high‐risk groups before wild mushrooms mature every summer and autumn. In addition, we recommend that ordinary people not pick wild mushrooms outdoors for consumption, because it is difficult to distinguish whether wild mushrooms are poisonous and do not buy wild mushrooms of unknown sources.

## Graphical abstract

- There were seasonal, high‐incidence areas and high‐risk groups in wild mushroom poisoning.
- The incidence of mushroom poisoning in Zhejiang province was high in households (91.6%), mainly in rural households.
- Local spatial autocorrelation showed that the “hot spots” of wild mushroom poisoning in Zhejiang province from 2016 to 2018 were Quzhou and Lishui.

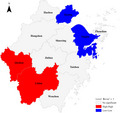

## 1 INTRODUCTION

Mushrooms are macroscopic fleshy mostly edible fungi that grow from mycelia. They have the ability to grow on a wide variety of substrates and are widely used in food, medicine, and religious rituals. There are about 14,000 species of mushrooms known in the world, and more than 4,000 species known are estimated in China, among which 936 are edible fungi (Dai et al., 2010), 437 are medicinal fungi (Dai & Yang, 2008), and 435 species are poisonous mushrooms (Bau et al., 2014; Li et al., 2013). Poisonous mushrooms refer to the species that can produce toxic reactions to humans, livestock, and poultry after the fruiting bodies of large fungi are consumed (Mao, 2006). There were cases caused by the accidental ingestion of poisonous mushrooms every year around the world (Berch et al., 2017; Cervellin et al., 2017; Govorushko, 2012; Jo et al., 2014; Zhou et al., 2016). The main cause of poisoning is confusing edible mushrooms with poisonous mushrooms. Consumption of poisonous mushrooms can cause various signs and symptoms, such as gastroenteritis, disturbances in central nervous system, and liver failure. Even worse, if mushroom poisoning is not treated in time, it can lead to death (Diaz, 2005).

China has a long history of collecting and eating wild mushrooms. As a result, there are a large number of cases of wild mushroom poisoning every year. According to the monitoring data, we find that mushroom poisoning is the leading cause of death from foodborne disease outbreaks in Zhejiang province (Sun, Chen, et al., 2018). Therefore, preventive measures must be taken to reduce the occurrence of mushroom poisoning. In this study, we collected the data of reported cases of wild mushroom poisoning in Zhejiang province from 2016 to 2018 and conducted a descriptive analysis to explore the epidemiological characteristics, aiming to put forward targeted prevention and control measures.

## 2 MATERIALS AND METHODS

### 2.1 Data sources

The data came from “Zhejiang Province Foodborne Disease Surveillance and Reporting System,” which has collected sporadic cases of foodborne diseases (including infectious cases and poisoned cases) from 340 sentinel hospitals in 11 prefectural cities. All data about the wild mushroom poisoning cases reported from January 2016 to December 2018 were included. The diagnosis of wild mushroom poisoning is based on epidemiological investigation, history of eating wild mushroom, and clinical manifestations. The address data of 429 cases were transformed to geographic spatial data based on the Baidu geocoding API (http://lbsyun.baidu.com/index.php?title=webapi/guide/webservice‐geocoding).

Population data of prefectures and counties from 2016 to 2018 are obtained from Zhejiang Provincial Statistics Bureau. The GIS map data of Zhejiang province is downloaded by the national basic geographic information center of China (http://ngcc.sbsm.gov.cn).

### 2.2 Statistical methods

#### 2.2.1 Descriptive analysis

The following parameters were extracted (if available) from the charts of included patients: age, sex, hospitalization, occupation, home address, time of onset, time of visit, time of death, main symptoms and signs, method of acquiring poisonous mushroom, type of dining place, and time of eating. Data were analyzed descriptively using the software of GraphPad Prism version 9.2.0 (GraphPad Software, 2021).

#### 2.2.2 Production of spatial distribution map

We used ArcGis10.2 software to connect case database (including geocoding, number of cases, incidence, etc.), geographical information database (including geocoding, dimension and longitude) with population information database (including geocoding, population) through geocoding connection, established cases geographic information database in Zhejiang province, and then made a space distribution.

#### 2.2.3 Spatial autocorrelation analysis

Spatial autocorrelation is a measure of the degree to which one thing or phenomenon is similar to other nearby things or phenomena, which includes global autocorrelation and local autocorrelation. Global autocorrelation describes the degree of association between spatial objects in the whole research area to indicate whether there is a significant spatial distribution pattern between spatial objects. A well‐known spatial statistic for measuring spatial autocorrelation is Moran’s *I* (Moran, 1948). It was defined as follows:

(1) $$I=\frac{N}{S_{0}}\;\frac{\sum_{i}^{n}\sum_{j=1}^{n}w_{\mathrm{ij}}\left(x_{i}‐\mu \right)\left(x_{j}‐\mu \right)}{\sum_{i}{\left(x_{i}‐\mu \right)}^{2}}$$

where *N* is the number of regions, *x_i_* is the value of attribute *X* (e.g., incidence of mushroom poisoning) at region *i*, *μ* is the mean value of *X*, and *w_ij_* is a matrix indicating the spatial weight between regions *i* and *j*. *S*_0_ is the sum of the weight calculated by:

(2) $$S_{0}=\sum_{i}\sum_{j}w_{\mathrm{ij}}$$

Moran’s *I* value is between −1 and +1, and the value is positive, indicating a positive correlation with the data. The closer the value is to 1, the stronger the positive spatial correlation is, and the case occurrence space presents an aggregation distribution. Moran’s *I* was negative, and the data were negatively correlated. The closer to −1, the more scattered the cases were, and the greater the difference between samples was. Moran’s *I* is 0, indicating that cases occur randomly in space. However, the global autocorrelation only reflects the spatial correlation of the research target. To understand the correlation (positive and negative, as well as the degree of correlation) between a district and surrounding counties, we need to use the local Moran’s *I* coefficient. Local autocorrelation analysis is used to test the probability level of local spatial aggregation around each observation unit. When there is no global spatial autocorrelation, local autocorrelation analysis is used to find the location of local spatial autocorrelation that may be masked. When there is global spatial autocorrelation, local autocorrelation analysis is used to investigate whether there is spatial heterogeneity.

To explore the possible reasons behind the spatial patterns of mushroom poisoning, we calculated the global Moran’s *I* and local Moran’s *I* of the incidence of mushroom poisoning. ArcGIS software was used for the analysis. Weight matrix W was defined by the “contiguity” method, which only includes neighboring polygons for computation.

## 3 RESULTS

### 3.1 Basic information

From 2016 to 2018, 429 cases (244 males and 185 females) of wild mushroom poisoning were reported in Zhejiang province. Among them, there were 2 deaths (1 male, 1 female) and 84 hospitalizations, with the incidence of 0.2526 per 100,000 and the case fatality rate of 0.47%. In the past three years, cases of wild mushroom poisoning have increased annually, with all the deaths occurring in 2016 (Table 1). All wild mushroom poisonings occurred accidentally, resulting from consuming poisonous mushrooms collected from the wild or purchased from local markets. Digestive symptoms were found in all cases. Systemic symptoms and signs were found in 28.90% (124/429) of the cases, while neurological symptoms were 11.66% (50/429) and urinary symptoms were 4.90% (21/429). The proportion of cases with an incubation period of less than 6 h was 85.78%, and the cases with an incubation period of ≥6 h accounted for 14.22%. The incubation period of the 2 deaths was ≥6 h.

**TABLE 1:** Basic information of wild mushroom poisoning in Zhejiang province during 2016–2018

Year	*n*(%)		
	Poisoning	Hospitalizations	Deaths
2016	87 (20.28)	24 (28.57)	2 (100.00)
2017	106 (24.71)	18 (21.43)	0 (0.00)
2018	236 (55.01)	42 (50.00)	0 (0.00)
Total	429 (100.00)	84 (100.00)	2 (100.00)

### 3.2 Regional distribution

**FIGURE 1:**
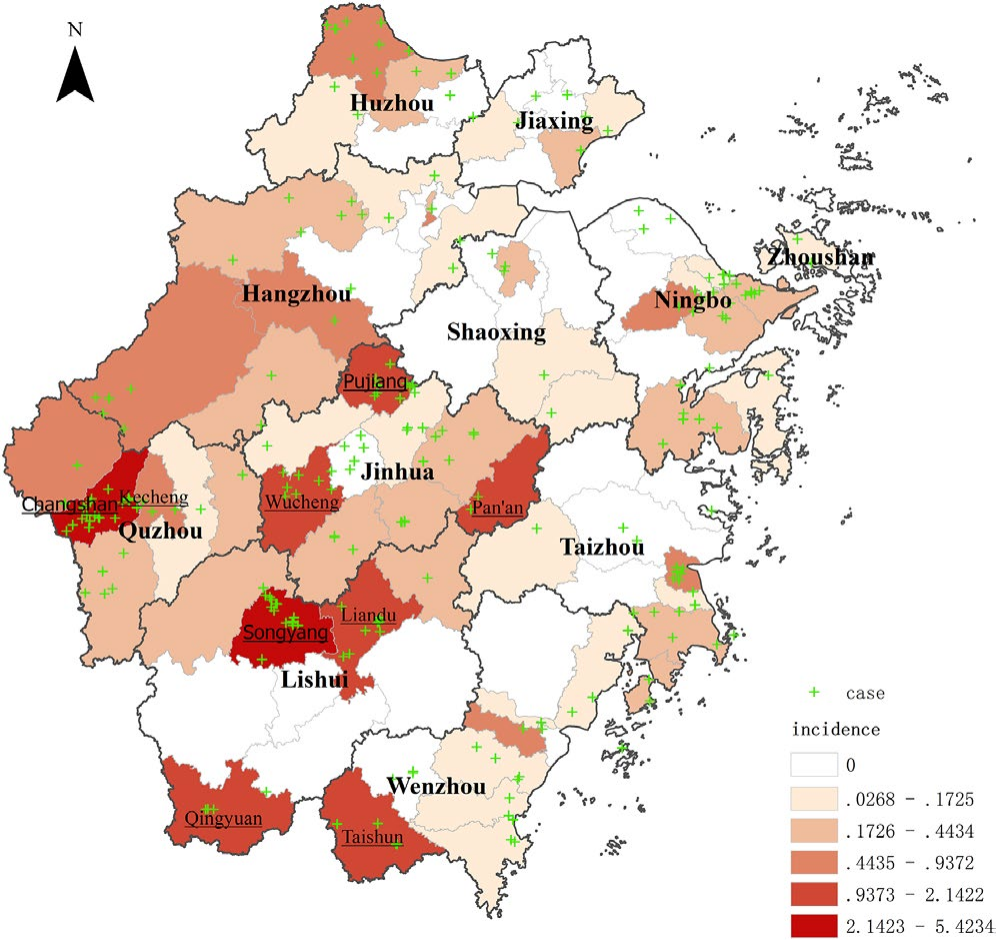
Incidence statistics of toxic mushroom poisoning in various counties of Zhejiang province from 2016 to 2018

The highest number of cases of mushroom poisoning occurred in Jinhua, followed by Quzhou and Lishui. The highest incidence was reported in Quzhou and the lowest in Zhoushan (Table 2). The counties with higher incidence were Songyang county (5.4234/100,000), Changshan county (5.3149/100,000), and Pujiang county (2.1422/100,000) (Figure 1).

**TABLE 2:** Incidence and number of cases of wild mushroom poisoning in Zhejiang province, by prefecture and year, 2016 to 2018

Prefecture	*N*				Reported incidence (/100,000)
	2016	2017	2018	2016–2018	
Quzhou	6	13	46	65	0.9915
Lishui	6	14	36	56	0.8550
Jinhua	22	19	58	99	0.5932
Huzhou	0	6	14	20	0.2223
Ningbo	26	13	10	49	0.2035
Taizhou	7	9	15	31	0.1691
Wenzhou	9	16	20	45	0.1628
Hangzhou	8	14	16	38	0.1335
Shaoxing	2	0	15	17	0.1131
Jiaxing	0	2	6	8	0.0572
Zhoushan	1	0	0	1	0.0286
Total	87	106	236	429	0.2526

### 3.3 Time distribution

**FIGURE 2:**
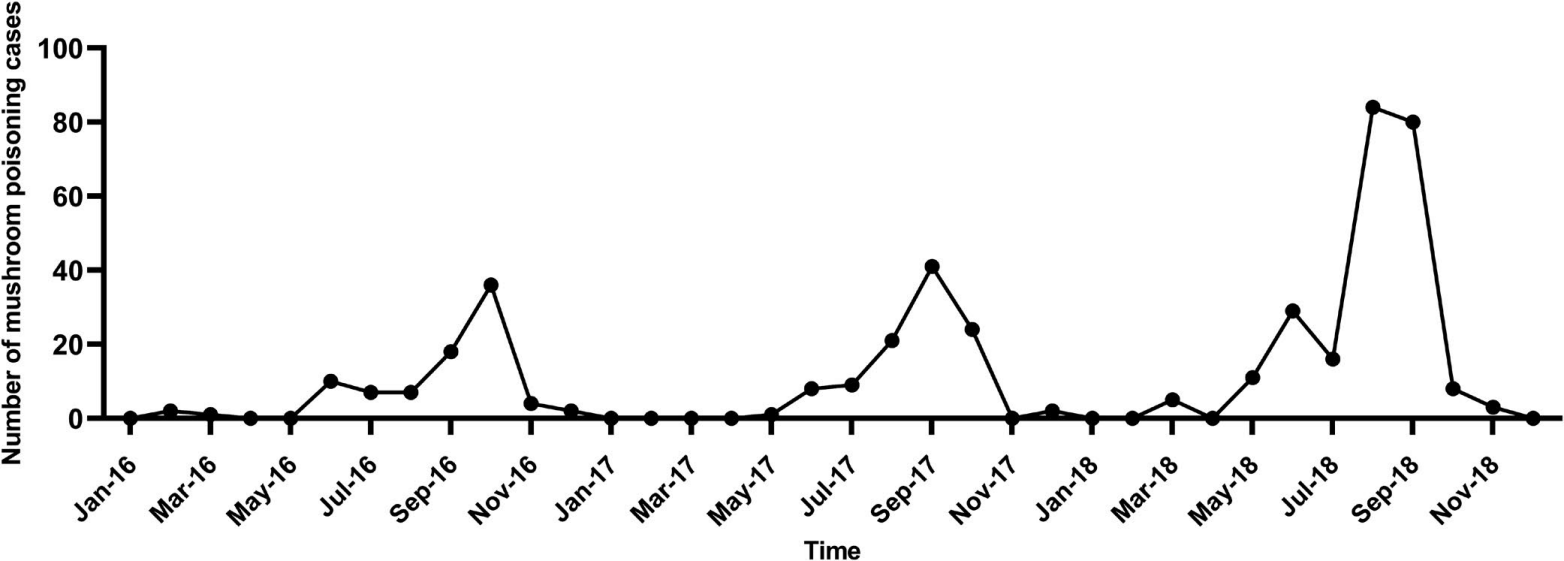
Time series of wild mushroom poisoning cases in Zhejiang province from 2016 to 2018

The occurrence of mushroom poisoning had obvious seasonality. Mushroom poisoning occurred most frequently from June to October, during which 373 cases occurred, accounting for 86.9% of the total cases (Figure 2).

### 3.4 Population distribution

Poisoning cases ranged in age from 1 to 88 years, with a median age of 46 years. Cases aged 26–60 years accounted for the largest proportion (68.30%). The occupation with the highest incidence rate was farmers (48.72%), followed by migrant workers (13.52%) and workers (7.23%). We found that 37 (8.62%, 37/429) poisoning cases were from other provinces in China, and the rest were residents of Zhejiang province.

### 3.5 Distribution of poisoning places

Most cases of mushroom poisoning occurred in households (91.61%), and the main sources of mushrooms were picking (93.94%) or buying (6.06%) wild mushrooms (Table 3). A small amount of mushroom poisoning occurred in collective canteens (5.59%), and the poisonous mushrooms mainly came from picking (62.5%) or buying (29.17%) wild mushrooms.

**TABLE 3:** Classification of wild mushroom poisoning sites in Zhejiang province during 2016–2018

Poisoning places	The number of poisoning (%)	The number of deaths (%)
Household	393 (91.61)	2 (100.00)
Collective canteens	24 (5.59)	0 (0.00)
Unknown	12 (2.80)	0 (0.00)

### 3.6 Global spatial autocorrelation analysis

The global Moran’s *I* of average incidence of mushroom poisoning was 0.337 (*z* = 2.37, *p* = .02 < .05), suggesting that the incidence distribution of wild mushroom *poisoning* in Zhejiang province during 2016–2018 was not random, but had a statistically significant spatial positive correlation, indicating a significant aggregation distribution (Table 4). The global Moran’s *I* was 0.087, almost 0, it indicated that the cluster effect of mushroom poisoning is weaker and shows a discrete and random distribution in 2016, while in 2017 and 2018, the global Moran’s *I* increased and the value showed a sped up aggregation tendency. By 2018, the global Moran’s *I* was 0.274, and the corresponding Z score was 2.216, *p* value = .027 < .05. It indicated that the aggregation trend of the incidence of mushroom poisoning was significant.

**TABLE 4:** Cluster tendency of incidence of mushroom poisoning from 2016 to 2018

Year	2016	2017	2018	2016–2018
Global Moran’s *I*	0.087	0.271	0.274	0.337
*Z* score (*p* value)	1.01 (.312)	2.132 (.033)	2.216 (.027)	2.513 (.012)

### 3.7 Local spatial autocorrelation analysis

**FIGURE 3:**
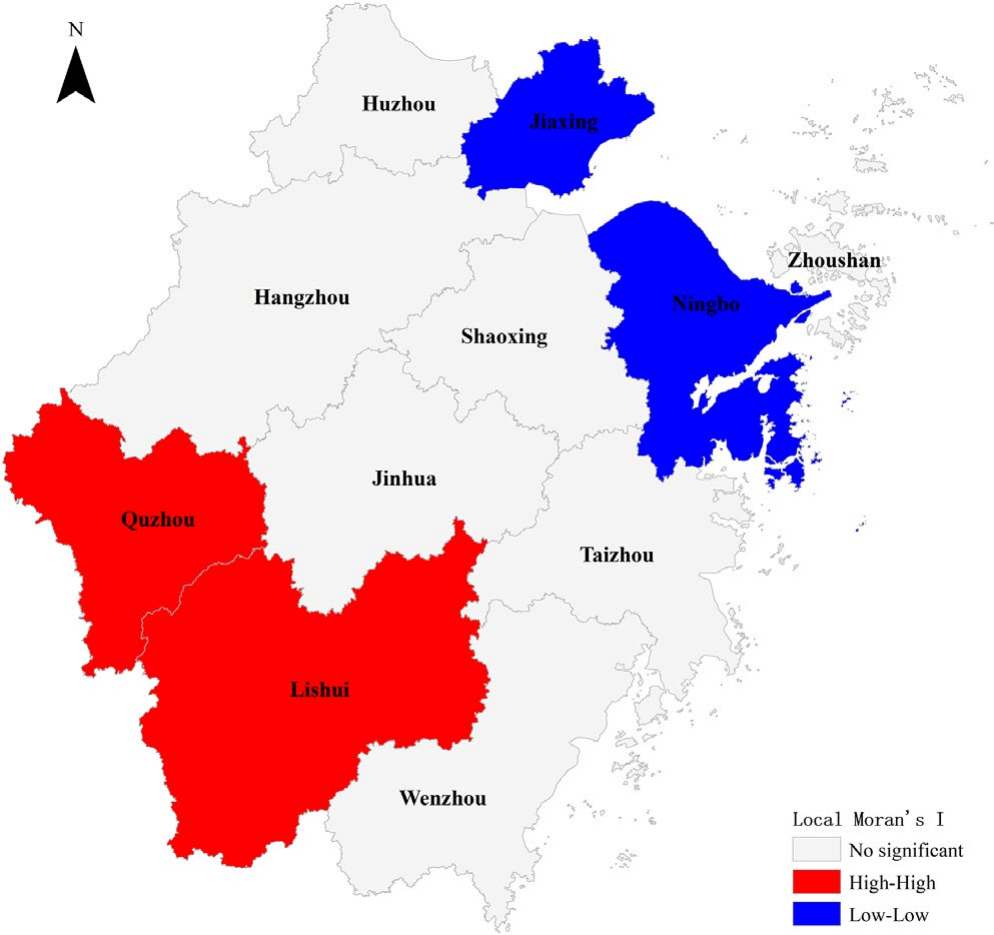
Spatial autocorrelation based analysis of incidence aggregation in different prefectures of Zhejiang province

There are 11 prefecture‐level cities in Zhejiang province. The results of local autocorrelation analysis are shown in Figure 3 and Table 5. The red area represents the high‐high spatial correlation model, which is mainly distributed in Quzhou and Lishui. The area with high incidence of wild mushroom poisoning is surrounded by high incidence, showing a state of high value aggregation (*p* < .05). It was a “hot spot” region for the incidence of wild mushroom poisoning in Zhejiang province from 2016 to 2018. The blue area represented the low‐low spatial correlation model, which is mainly distributed in Jiaxing and Ningbo. The area with low incidence of wild mushroom poisoning was surrounded by low incidence, showing a state of low value aggregation (*p* < .05). It was a “cold spot” region for the incidence of wild mushroom poisoning. The autocorrelation characteristics of other regions were not significant. The analysis of local Moran’s *I* showed that not only the high‐incidence area of poisonous mushroom should arouse the concern, but also the neighborhood area of the high value area should be paid more attention as potential high‐incidence areas.

**TABLE 5:** Results of local autocorrelation analysis on the incidence of wild mushroom poisoning in different cities of Zhejiang province during 2016–2018

Prefectures	Moran’s I	Association types	*p*‐ value
Hangzhou	−0.1254	0	.272
Ningbo	0.2413	2	.02
Wenzhou	−0.2712	0	.259
Jiaxing	0.4278	2	.047
Huzhou	0.1996	0	.135
Shaoxing	0.1671	0	.201
Jinhua	0.3216	0	.082
Quzhou	1.2423	1	.027
Zhoushan	0.5	0	.145
Taizhou	−0.0881	0	.325
Lishui	0.7588	1	.045

Notes

1, high‐high; 2, low‐low; 3, low‐high.

## 4 DISCUSSION

The incidence of mushroom poisoning varies worldwide. The incidence of mushroom poisoning is higher in countries where wild mushrooms have traditionally played an important role in food, such as Russia, Eastern Europe, and China (Govorushko et al., 2019). Yunnan and Guizhou in China had the highest mortality rates (Sun, Li, et al., 2018). Asian countries such as Nepal (Adhikari et al., 2005), Thailand (Parnmen et al., 2016), and Iran (Soltaninejad, 2018) also have high mortality rates. By contrast, deaths induced by mushrooms are rarely reported in the United States (Kintziger et al., 2011), Canada (Fleury et al., 2008), and Australia (Roberts et al., 2013), probably due to better health care. Although the incidence and mortality of mushroom poisoning in Zhejiang province were lower than those in some other provinces of China, such as Hunan (Liang et al., 2018), Yunnan (Wang et al., 2016), and Guangxi (Jiang et al., 2018), mushroom poisoning has become the main cause of death from foodborne disease outbreaks (Sun, Chen, et al., 2018), which does require more attention from relevant departments. One of the reasons for the high mortality rate of mushroom poisoning in China is that people in mountainous areas lack the ability to identify highly toxic mushrooms. On the other hand, many hospitals lack experience in the treatment of highly toxic mushrooms and often miss the best treatment period. Therefore, it is very important to strengthen the publicity of mushroom poisoning and the training of doctors. In addition, good communication between mushroom experts, epidemiologists, and doctors should be established to save cases of highly toxic mushroom poisoning.

In Diaz’s review (Diaz, 2005), 14 major syndromes of mushroom poisoning were stratified by presentation timing and then by target organ systemic toxicity and included early (<6 h, 8 subgroups), late (6–24 h, 3 subgroups), and delayed syndromes (>1 day, 3 subgroups). Since the incubation period after consumption of wild mushrooms can provide useful information for clinicians to understand the severity and prognosis of wild mushroom poisoning, we collected the incubation period for all cases. In our study, a total of 85.78% of poisoning cases belonged to the early type (<6 h) and the prognosis was good. The incubation periods of the two deaths were 6 and 9 h, respectively, and their initial symptoms were gastrointestinal symptoms, but they later died of liver and kidney failure.

Findings showed that the incidence of mushroom poisoning in Zhejiang province was high from June to October, indicating that prevention and control should be strengthened in summer and autumn, and an early warning should be issued before poisonous mushrooms mature. The incidence of mushroom poisoning in Zhejiang province was high in households (91.6%), mainly in rural households, which was consistent with some studies at home and abroad (Chen et al., 2019; Chibishev et al., 2015; Durukan et al., 2007; Karahan et al., 2016; Verma et al., 2014; Wang et al., 2014; Zhou et al., 2016). The poisonous mushrooms were mostly harvested by farmers themselves. Farmers or workers who had the habit of eating wild mushrooms but did not have the ability to identify poisonous mushrooms were most likely to suffer from wild mushroom poisoning. Therefore, it is necessary to strengthen publicity among high‐risk groups. The identification of poisonous mushrooms is mainly based on the external morphology, microscopic characteristics, and DNA molecular markers identified by experts (Apperley et al., 2013; Chen, 2014). It is difficult for the average person to distinguish edible mushrooms from poisonous ones, so it is advised not to pick wild mushrooms outdoors for consumption.

In epidemiological studies, the distribution of most diseases is not random, but aggregated to a certain extent. “Hot spots” of disease incidence mean that there are clusters in this region and the clusters are areas with high incidence. Exploring “hot spots” can provide scientific basis for disease prevention and control strategies and measures. In our study, local spatial autocorrelation showed that the “hot spots” of wild mushroom poisoning in Zhejiang province from 2016 to 2018 were Quzhou and Lishui. The two areas are characterized by hills and mountains, high forest coverage, and abundant rainfall. The incidence of mushroom poisoning was lower in Jiaxing, a city mainly on the plain, and Ningbo, a coastal city, which may be related to the growing environment of wild mushrooms and the eating habits of local residents. In the prevention and control of mushroom poisoning, attention should be paid to the early warning, publicity, and supervision of “hot spot” areas.

## 5 CONCLUSIONS

Based on epidemiological analysis, we found that there were seasonal, high‐incidence areas and high‐risk groups in wild mushroom poisoning. Therefore, in order to effectively reduce the incidence of mushroom poisoning, it is suggested that the government should give early warning to high‐incidence areas and strengthen publicity to high‐risk groups before wild mushrooms mature every summer and autumn. In addition, we recommend that ordinary people not pick wild mushrooms outdoors for consumption, because it is difficult to distinguish whether wild mushrooms are poisonous and do not buy wild mushrooms of unknown sources.

## CONFLICTS OF INTEREST

The authors declare no conflict of interest.

## AUTHOR CONTRIBUTIONS

**Lili Chen:** Conceptualization (equal); Data curation (equal); Methodology (equal); Writing‐original draft (equal). **Liang Sun:** Conceptualization (equal); Data curation (equal); Methodology (equal); Writing‐original draft (equal). **Ronghua Zhang:** Project administration (lead). **Ningbo Liao:** Investigation (lead). **Xiaojuan Qi:** Methodology (equal). **Jiang Chen:** Writing‐review & editing (equal). **Ting Liu:** Writing‐review & editing (equal).

## ETHICS APPROVAL

All procedures performed in studies did not involve human participants and animals. Permission to use the data from *Zhejiang Province Foodborne Disease Monitoring and Reporting System* was granted by Zhejiang Provincial Center for Disease Control and Prevention.

## Data Availability

The data used to support the findings of this study are available from the corresponding author upon request.
